# Patents on Technologies of Human Tissue and Organ Regeneration from Pluripotent Human Embryonic Stem Cells

**DOI:** 10.2174/2210297311101020142

**Published:** 2011-05

**Authors:** Xuejun H Parsons, Yang D Teng, Dennis A Moore, Evan Y Snyder

**Affiliations:** 1 San Diego Regenerative Medicine Institute, San Diego, CA 92121, USA; 2 Xcelthera, San Diego, CA 92121, USA; 3 Department of Neurosurgery, Harvard Medical School, and Division of SCI Research, VA Boston Healthcare System, Boston, MA 02115, USA; 4 Program in Stem Cell & Regenerative Biology, Sanford-Burnham Medical Research Institute, La Jolla, CA 92037, USA

**Keywords:** Human embryo, human embryonic stem cell patent, derivation, differentiation, cell culture, human embryonic stem cell, human pluripotent stem cell, human neural stem, progenitor, precursor cell, human somatic stem cell, human stem cell, pluripotence, multipotence, neuron, oligodendrocyte, retinal pigment epithelium cell, cardiomyocyte, endoderm cell, pancreatic cell, hematopoietic cell, hepatocyte, connective tissue progenitor, osteoblast and chondrocyte precursor, mesenchymal stem cell, cell therapy, regenerative medicine.

## Abstract

Human embryonic stem cells (hESCs) are genetically stable with unlimited expansion ability and unrestricted plasticity, proffering a pluripotent reservoir for in vitro derivation of a large supply of disease-targeted human somatic cells that are restricted to the lineage in need of repair. There is a large healthcare need to develop hESC-based therapeutic solutions to provide optimal regeneration and reconstruction treatment options for the damaged or lost tissue or organ that have been lacking. In spite of controversy surrounding the ownership of hESCs, the number of patent applications related to hESCs is growing rapidly. This review gives an overview of different patent applications on technologies of derivation, maintenance, differentiation, and manipulation of hESCs for therapies. Many of the published patent applications have been based on previously established methods in the animal systems and multi-lineage inclination of pluripotent cells through spontaneous germ-layer differentiation. Innovative human stem cell technologies that are safe and effective for human tissue and organ regeneration in the clinical setting remain to be developed. Our overall view on the current patent situation of hESC technologies suggests a trend towards hESC patent filings on novel therapeutic strategies of direct control and modulation of hESC pluripotent fate, particularly in a 3-dimensional context, when deriving clinically-relevant lineages for regenerative therapies.

## INTRODUCTION

The successful derivation of human embryonic stem cell (hESC) lines from the *in vitro *fertilization (IVF) leftover embryos in 1998 is considered as one of the major breakthroughs of the 20th century life sciences [1]. The hESCs, derived from the inner cell mass (ICM) or epiblast of human blastocyst, are genetically stable with unlimited expansion ability and unrestricted plasticity, proffering a pluripotent reservoir for *in vitro* derivation of a large supply of disease-targeted human somatic cells that are restricted to the lineage in need of repair [1, 2]. Therefore, they have been regarded as an ideal source to provide an unlimited supply of large-scale well-characterized human specialized cell types for cell-based therapies to resolve some worldwide major health problems, such as neurodegenerative diseases, paralysis, diabetes, and heart diseases. Most recently, the IVF pioneer Robert Edwards was awarded 2010 Nobel Prize in physiology or medicine [3]. The Noble prize recognition to the IVF techniques comes to light that a small portion of the millions of excess embryos currently stored in the IVF clinics worldwide, which are otherwise destined for destruction, could potentially be an unlimited source to deliver in the future a whole range of therapeutic treatments for tissue and function restoration in patients with life-threatening diseases and injuries.

The traditional sources of engraftable human stem cells for transplantation therapies have been multipotent human somatic stem cells (hSSCs) isolated directly from the tissue or organ system of interest [2, 4, 5]. However, cell therapies based on tissue-derived hSSCs have encountered supply restriction and difficulty to use in the clinical setting due to their limited expansion ability in culture and failing plasticity after extensive passaging [2, 4, 5]. The propagation ability of such tissue-derived hSSCs is often limited, making it difficult to establish a large scale culture. Their transplantation efficiency and plasticity further decline after extensive culture. Despite some beneficial outcomes, the small numbers of functional progenies generated from engrafted tissue-derived hSSCs often fail to achieve the anticipated mechanism of direct reconstruction of the damaged structure and circuitry [2, 4, 5]. So far, due to these major limitations, cell therapies based on tissue-derived hSSCs have not yielded the satisfactory results expected for clinical trials to move forward. Alternatively, pluripotent hESCs have the capacity for long-term undifferentiated growth in culture, as well as the theoretical potential for differentiation into any cell type in the human body [1, 2]. These properties offer hESCs as a potential unlimited source for transplantation therapies and as a model system for studying mechanisms underlying human development. The hESCs and their derivatives are considerably less immunogenic than adult tissues [2]. It is also possible to bank large numbers of human leukocyte antigen isotyped hESC lines so as to improve the likelihood of a close match to a particular patient in order to minimize the potential risk and side-effect of immune rejection following transplantation. However, recent court battle on Federal funding for hESC research in the United State (US) has highlighted the decade long social and legal controversy surrounding hESC research. As a result of policy battles concerning hESC research, artificially-reprogrammed somatic cells -- the induced pluripotent stem cells (iPSCs) -- were created by over-expression of proliferative embryonic genes in adult cells in order to circumvent the ethical issues associated with the derivation of hESCs [6-9]. Although it may provide patient-specific pluripotent cells to avoid immune rejection, the iPSC technique is extremely time-consuming and inefficient in restoring an embryonic state. Unlike tightly-regulated *in vivo* biological reprogramming in the human reproduction process, insertion or transient expression of foreign oncogenes in adult somatic cells at a non-physiological level tends to induce cancer phenotypes and malignant transformation of iPSCs, resulting in low survival rates and genetic-defects of iPSC-derived fetus [10]. The iPSCs are characterized by expressing embryonic markers that are initially identified in embryonic tumor/cancer cells and forming teratomas *in vivo* without the evidence for maintaining long-term genetic integrity and stability [6-9]. In fact, the iPSCs differ dependent on cell type of origin and display abnormal gene silencing of somatic cells, and iPSC-derived cells show accelerated senescence [11-13]. These major drawbacks severely impair the reprogrammed iPSCs’ clinical utility. So far, the hESCs remain as the most genetically-stable human pluripotent cell source with full-developmental potential in deriving somatic elements for tissue and function restoration. 

US patents directed to human stem cell technologies have generated intense interest as well as controversy [14-16]. Many patents relating to stem cell technology have faced reexamination, litigation, or both [17, 18]. The US Patent and Trademark Office (USPTO) recently upheld three Wisconsin Alumni Research Foundation (WARF) stem cell patents, US5843780 (1998), US6200806 (2001), and US7029913 (2006), on the breadth, anticipation, and obviousness of the claims after reexamination requested by a third-party challenger in 2006 [17, 18]. These WARF patents involve claims on hESCs as well as certain processes used to make such cells as divisional applications originated from primate embryonic stem cell patents [19, 20]. These WARF patents with extremely broad claims have casted a shadow over the commercialization of these cells as therapeutics in the US’ biotechnology market so far [20]. While the controversies related to hESC patents in the US center on scientific and economic issues, in Europe, the patentability of hESCs has been met with fierce moral opposition [14]. The European Patent Office (EPO) has refused to grant hESC patents based on its interpretation of the “European Directive on the Legal Protection of Biotechnological Inventions”, which holds unpatentable inventions concerning products of human stem cell cultures that can only be obtained by the use, involving their destruction, of human embryos [14, 21-25]. The EPO regards patents on hESCs as illegal because they are patents on a human body or human body part, offend human dignity, or involve commercial or industrial uses of embryos [14, 21-25]. However, in spite of controversy surrounding the ownership of hESCs, the number of patent applications related to hESCs is growing rapidly in the last 5 years. It will be of importance to fulfilling the therapeutic promise of hESCs that the hESC patents are placed in the context of the biotechnology and health industry and granted on inventions downstream in the value chain of regenerative medicine [26, 27]. To date, the USPTO has granted 92 hESC-related patents on the process for isolating, culturing, purifying, manipulating, or differentiating hESCs.

## DERIVATION OF hESCs

The hESC lines were initially derived from the ICM of human blastocysts using growth-arrested mouse embryonic fibroblasts (MEF) as feeder layers to maintain long-term undifferentiated growth in culture [1, 28]. Although the hESC lines were isolated in 1998, the WARF was able to claim the hESC patent first by filing a divisional patent application to their previously-granted primate embryonic stem cell (ESC) patent US5843780 (1998), which discloses the primate animal cells of preparation characterized by the cell surface markers SSEA-1 (-), SSEA-3 (+), SSEA-4 (+), TRA-1-60 (+), TRA-1-81 (+), and alkaline phosphatase (+) and a method for isolating a primate ESC line that are basically identical to mouse ESCs and their isolating process [20, 28]. The USPTO granted the WARF’s claims on hESCs and the method used to make such cells in 2001 (US6200806), and a continuation of claims on hESCs in 2006 (US7029913), which are currently under reexamination [17-20, 28]. The WARF preparation of hESCs is characterized by the cell surface markers SSEA-1 (-), SSEA-4 (+), TRA-1-60 (+), TRA-1-81 (+), and alkaline phosphatase (+), normal karyotypes, continue to proliferate in an undifferentiated state after continuous culture for eleven months, and retain the ability to form trophoblast and to differentiate into all tissues derived from all three embryonic germ layers (endoderm, mesoderm and ectoderm) [28]. A continuation of the WARF’s claims on this preparation of hESCs for the process of spontaneously differentiated human cells *in vitro* in the absence of a fibroblast feeder layer such that the cells differentiate into human endoderm cells was granted in 2010 (US7781216) [28] Table **1**. 

The ES Cell International in Singapore owns US6875607 patent (2005) on invention related to undifferentiated hESCs, methods of cultivation and propagation, production of differentiated cells and in particular the production of committed progenitor cells capable of giving rise to mature somatic cells and uses thereof, and a purified preparation of undifferentiated hESCs capable of proliferation *in vitro* [29] Table **1**. The US7153684 patent (2006) of Vanderbilt University in the US claims invention on pluripotential non-mouse ESCs, including human, that can be maintained on feeder layers and give rise to embryoid bodies and multiple differentiated cell phenotypes in monolayer culture, and a method of making a pluripotential ESC comprising culturing germ cells and germ cell progenitors in growth enhancing factors of basic fibroblast growth factor (bFGF), leukemia inhibitory factor (LIF), and steel factor [30] Table **1**. The Reliance Life Sciences in India claims a method for isolating an ICM from the blastocyst stage embryo by laser ablation (US7294508, 2007) to establish hESC lines (US7811817, 2010) [31, 32] Table **1**. The US6921632 patent (2005) of the Maria Biotech in Korea covers a method for establishing undifferentiated hESCs by thawing cryopreserved human embryos, preferably blastocyst stage embryos, and culturing at least a portion of the embryos on a medium capable of sustaining undifferentiated hESCs [33] Table **1**. Most recently, the successful derivation of hESCs from earlier morula (eight-cell)-stage embryos has resulted in patent applications on such inventions [34-36] Table **1**. Development of techniques for derivation of hESC lines from floating ICMs in suspension culture conditions that do not involve feeder cells or microcarriers may pave the way for large-scale expansion and controlled differentiation of hESCs in suspension, which would be valuable in basic and applied research in regenerative medicine [37, 38] Table **1**.

Our lack of knowledge regarding the essential components required for the growth and maintenance of hESCs in an undifferentiated state, however, remains as a major obstacle for clinical translation of this potentially powerful biology. To compensate for this gap in our knowledge, undefined biological supplements and/or feeder layers have typically been used for the isolation, expansion, maintenance, and differentiation of hESCs. Ironically, it is the very need for these foreign biologics that may make direct use of these hESCs and their derivatives in patients problematic. Historically, hESCs were derived and maintained in co-culture with mouse feeder cells or their conditioned media [1, 28, 39]. Using this mouse-support system may compromise the therapeutic potential of these cells because of the risk of transmitting xenopathogens, altering genetic background, and promoting the expression of immunogenic proteins [40, 41]. Without an understanding of the essential components for sustaining pluripotence and self-renewal of hESCs, such cell lines isolated and expanded in claimed artificially-formulated ingredients (WARF patent US7442548, 2008) remain at risk for becoming unhealthy and genetically unstable after prolonged culturing [42, 43] Table **1**.

## CULTURE TECHNIQUES AND PLURIPOTENCE MAINTENANCE OF hESCs

### Xeno-free Feeder Layer for the Growth of Undifferentiated hESCs

Maintaining hESCs in an undifferentiated state such that they can be repeatedly and reliably expanded is one of the keys to their utility and potential. Achieving this goal, however, likely requires a better knowledge of the essential requirements for maintaining the undifferentiated state of pluripotent cells. The hESC lines initially were derived and maintained in co-culture with growth-arrested MEFs as feeder layers or MEF-conditioned media on substrata such as laminin or laminin-collagen combinations (commercially known as Matrigel) [1, 28, 39, 44-46] Table **2**. Using this mouse-support system may compromise the therapeutic potential of hESCs because of the risk of transmitting pathogens and altering genetic background from the animal cells. Recognizing this limitation, some investigators have shown that undifferentiated hESCs can be cultivated on human feeder layers, including human fetal and adult fibroblast feeders, human foreskin fibroblast feeders, and human bone marrow stromal cells [40, 47-51] Table **2**. This approach addresses the risk of transmitted xenopathogens, though the risk of transmitted human pathogens or other potential contaminants remains. In order to establish a culture system that was free of any animal products, we have tested the human foreskin fibroblast cell line (ATCC cell line Hs27) as a human feeder layer for the growth of undifferentiated hESCs at the beginning [52]. In the first attempts to transfer the hESCs to the human feeder layers, we observed far more differentiated cells compared to those grown on MEFs. The hESC colonies maintained on human feeders displayed a more irregular morphology, more elliptical and less round, and considerably smaller than those grown on MEFs. Surprisingly, we discovered that, by increasing the bFGF concentration in the hESC medium to 20ng/ml (from 4ng/ml), the hESC colonies grown on the human cells displayed the more round and undifferentiated morphology, and significantly larger, suggesting that bFGF promoted undifferentiated growth of hESCs on feeder layers [52] Fig. (**1**). In addition to bFGF (20ng/ml), the medium used to obtain these results contained 80% DMEM/F-12, 20% knockout Serum Replacement, L-alanyl-L-glutamine (2 mM), MEM nonessential amino acids (1X), and β-Mercaptoethanol (100µM) [52]. In this media, >80% undifferentiated hESC colonies were now observed on the human feeders on every passage. Using this system, we have maintained undifferentiated hESCs on human feeder layers for over 12 months (>50 passages), thereby exhibiting sustained long-term stable undifferentiated growth as assessed both by morphological and immunological criteria and pluripotence as assessed by teratoma formation [52]. Specifically, hESCs maintained on human feeders displayed uniform undifferentiated morphology as well as high expression levels of Oct-4, SSEA-4, Tra-1-60, and Tra-1-81, but not SSEA-1 Fig. (**1**). The hESC colony is a dynamic structure that displays spontaneous early differentiation processes. Cells at the edge of the colonies exhibited the classic signs of early differentiation: flat epithelial cell-like morphology; expression of the cell surface marker SSEA-3 and the neural/beta-cell precursor marker Nestin Fig. (**1**). Cells that migrated beyond the edge of the colonies continued to differentiate further into large elliptical cells that persisted in expressing Nestin and appropriately now downregulated SSEA-3 Fig. (**1**).

### Feeder-Free Culturing of Undifferentiated hESCs

Alternatively, feeder layer-free culture systems have been suggested for hESCs [53-62] Table **2**. However, these feeder-independent culture systems require either feeder-conditioned media or artificially-formulated ingredients on matrix proteins in order to maintain the undifferentiated growth of hESCs [53-62] Table **2**. Culturing hESCs in feeder- and serum-free conditions appears to be sub-optimal for sustaining the undifferentiated state, so it is not clear that hESCs can be maintained in an undifferentiated state for long periods of time in these feeder-free systems [40, 42, 63]. Recent attempts to replace the undefined xenogeneic Matrigel with the purified human ECM proteins, serum matrices, or synthetic biomaterials as the matrix proteins also seem not up to the standards of long-term maintenance of hESC pluripotence [64-67] Table **2**. Long-term cultivation of undifferentiated hESCs in a biologics-free fully defined conditions -- i.e., feeder-, serum-, and conditioned-medium-free – will be crucial for providing an unlimited supply of well-characterized healthy cells for cell-based therapy, as well as for directing their lineage-specific differentiation.

### Defined Components for hESC Pluripotence Maintenance

Our inability to formulate totally-defined derivation and maintenance conditions for hESCs likely reflects a more fundamental gap in our knowledge regarding the minimal essential molecules and components necessary for the well-being and maintenance of undifferentiated hESCs, a platform from which differentiation can then proceed normally. Although several human feeder, feeder-free, and chemically-formulated culture systems have been developed for hESCs Table **2**, the elements necessary and sufficient for sustaining the self-renewal of hESCs remain unsolved. These exogenous feeder cells and biological reagents help maintain the long-term stable growth of undifferentiated hESCs whereas mask the ability of human pluripotent cells to respond to developmental signals. Maintaining undifferentiated hESCs in a defined biologics-free culture system that allows faithful expansion and controllable direct differentiation is one of the keys to their therapeutic utility and potential. A defined platform for the maintenance of pluripotent hESCs may overcome some of the major obstacles in translational biology, including *de novo* derivation of clinically-suitable hESCs and effectively directing such hESCs uniformly towards particular phenotypes. Maintaining pluripotent hESCs in a defined culture might enable the spontaneous unfolding of early embryogenic processes *in vitro* that emulate the *in vivo* maintenance of the pluripotent epiblast. In early embryogenesis, the epiblast is composed of more progressed pluripotent cells developed from the ICM, serving as the most immediate precursors of the early somatic lineages [68-70]. Therefore, such defined culture system might not only render specification of clinically-relevant early lineages directly from the pluripotent state without an intervening multi-lineage germ-layer stage, but also allow identify the signaling molecules necessary and sufficient for inducing the cascade of organogenesis in a process that may emulate the human embryonic development.

To resolve this quandary, we have identified the minimal essential requirements, including bFGF (20ng/ml), insulin, ascorbic acid, and laminin, to be both sufficient and necessary for the long-term stable growth of undifferentiated hESCs, recognizing that, in doing so, suggestions for an efficient totally-defined biologics-free culture system might emerge [52] Table **2**. We examined the growth of hESCs on Matrigel-coated plates in the defined hESC media containing 20ng/ml bFGF. Over 80% of hESC colonies maintained on Matrigel-coated plates in each passage were highly compact and undifferentiated as evidenced by their morphology and by their expression of Oct-4, SSEA-4, Tra-1-60, and Tra-1-81 by day 7 Fig. (**2**) [52]. The colonies on Matrigel had a more uniform morphology than those grown on human feeders, as indicated by the presence of an even narrower edge of SSEA-3-positive transitional imminently-differentiating cells Fig. (**2**) (compare to Fig. (**1**)). Undifferentiated hESC colonies have been maintained for over 8 months (>32 passages) on Matrigel-coated plates with normal karyotypes, suggesting that long-term stable undifferentiated growth of hESCs has been sustained. To further assess the effect of bFGF on hESC undifferentiated growth, we performed short-term proliferation assays of hESCs maintained under the feeder-free condition in the defined hESC media containing 0, 4, 10, 20, 30, or 50ng/ml bFGF. The growth rate and the percentage of undifferentiated colonies in response to bFGF doses were compared to those of hESCs maintained in MEF-conditioned media. In the defined media containing no bFGF or a low concentration of bFGF (4ng/ml), hESCs displayed significantly slow growth and high differentiation rates Fig. (**2**). In media supplemented with bFGF at a concentration ranging from 10 to 50ng/ml, hESCs displayed a growth rate comparable to that maintained in MEF-conditioned media, while the optimal proportion of undifferentiated hESCs appeared to be maintained at 20ng/ml bFGF Fig. (**2**) [52]. Our results suggest that bFGF is a critical component for sustaining undifferentiated growth and, at the proper concentration, may substitute for feeder cells or MEF-conditioned media Fig. (**2**) [52]. 

Having determined that substantial numbers of undifferentiated hESCs could be maintained over long periods in feeder-free environments using the bFGF-supplemented media, we further examined other components in the medium necessary to maintain hESCs in an undifferentiated state [52]. "Knockout Serum Replacement”, a semi-defined commercial additive, contains insulin, transferrin, ascorbic acid, amino acids, and AlbuMAX (a chromatographically-purified lipid-rich bovine serum albumin [BSA] with low IgG content, but nevertheless a xeno-derived product). Accordingly, we asked whether insulin, transferrin, BSA, and ascorbic acid were essential components, in combination with bFGF, for maintaining hESCs in an undifferentiated state. The serum replacement components insulin (20µg/ml), transferrin (8µg/ml), AlbuMAX (10mg/ml), and ascorbic acid (50µg/ml) were added to a base medium that consisted of 100% DMEM/F-12 with bFGF (20 ng/ml), L-alanyl-L-glutamine (2mM), MEM essential amino acids solution (1X), MEM nonessential amino acids solution (1X), and β-mercaptoethanol (100µM). To assay for the differen-tiation-forestalling activity of each of these components, undifferentiated hESCs were seeded on Matrigel- or human-laminin-coated plates and cultivated for seven days in media containing one or more of the individual components. When all of the components were present, >70% of the hESC colonies had a highly compact undifferentiated morphology and expressed Oct-4 Fig. (**3**), suggesting that these factors were sufficient to support undifferentiated growth of a substantial number hESCs [52]. In the absence of transferrin, fewer total hESC colonies were observed, but >70% of the hESC colonies that were present had a highly compact undifferentiated morphology and expressed Oct-4 Fig. (**3**). In the absence of AlbuMAX, hESC colonies were more flat and spread out, but >70% of the cells that were present nevertheless con-tinued to express Oct-4 and exhibited a highly compact undifferentiated morphology Fig. (**3**). However, if ascorbic acid was omitted from the media, the colonies often became very dense at their core and necrotic [Fig. (**3**) red arrows], suggesting that ascorbic acid was an essential component for maintaining the well-being as well as the undifferentiated growth of hESCs. When either bFGF or insulin was omitted from the media, >90% of the colonies appeared to differentiate completely within the first passage, as indicated by their differentiated morphology and their expression of the cell surface marker SSEA-1 Fig. (**3**). Conversely, undifferentiated hESCs maintained in media containing both bFGF and insulin did not express SSEA-1 Fig. (**3**). Large round cells were typically present in media that contained only insulin and elliptically-shaped cells were present in media that contained only bFGF Fig. (**3**), suggesting that insulin and bFGF might have distinct effects on hESC fate. The different effects of insulin and bFGF were accentuated further in media lacking ascorbic acid. In the absence of ascorbic acid and in media containing only insulin, the growth of differentiated hESCs was simply slower Fig. (**3**). In the absence of ascorbic acid and in media containing only bFGF, the appearance of cyst-like structures and necrotic cells within the dense cores of growing differentiated hESC colonies [Fig. (**3**), red arrow] became more severe. Taken together, these results suggested that, in addition to bFGF, insulin and ascorbic acid were also essential - perhaps in a collaborative manner - for maintaining substantial numbers of hESCs in a healthy undifferentiated state [52]. Although albumin and transferrin are not crucial components for sustaining the undifferentiated growth of hESCs, they might abet survival or maintain normal colony shape. We examined the effects other growth factors, including acidic fibroblast growth factor (aFGF), epidermal growth factor (EGF), insulin-like growth factor-I (IGF-I), insulin-like growth factor-II (IGF-II), platelet derived growth factor-AB (PDGF), vascular endothelial cell growth factor (VEGF), activin-A, and bone morphogenic protein 2 (BMP-2) on the growth of hESCs. In every case, most colonies (>70%) consisted of dense centers containing cyst-like structures and necrotic cells surrounded by a flat layer of fibroblast-like cells Fig. (**4A**). Although colony morphologies differed slightly depending on the growth factor used, none of the factors was sufficient for replacing bFGF in maintaining undifferentiated growth of hESCs. Interestingly, although most cells became differentiated when using these alternative growth factors, a minority of the small colonies (<30%) did retain compact morphologies and continued to express Oct-4 Fig. (**4A**). Having established that bFGF, insulin, and ascorbic acid were important minimal components of a feeder-free culture system, we further examined the growth of hESCs on purified matrix proteins, including human laminin-, fibronectin-, or collagen IV-coated plates in hESC media containing 20ng/ml bFGF [52]. Similar to hESCs maintained on Matrigel-coated plates, >80% of the hESC colonies remained undifferentiated on surfaces coated with laminin alone, as indicated by their classic undifferentiated morphology and their expression of Oct-4 Fig. (**4B**) [52], suggesting that the laminin portion of Matrigel is the critical component. In contrast, the majority of the hESC colonies (>70%) maintained on human fibronectin-, human collagen IV- , or, as a control, gelatin-coated plates, displayed a more differentiated morphology within the first passage, leaving only a minority (<30%) of small colonies bearing a compact, undifferentiated morphology Fig. (**4B**).

Interestingly, the colonies of undifferentiated cells maintained under the feeder-free conditions (on either laminin or laminin/collagen-coated plates) appeared to be associated with a monolayer of hESC-derived fibroblastic cells that expressed Nestin and Vimentin Fig. (**2**), suggesting that these cells may spontaneously act as “auto feeder layers” for the very same undifferentiated hESC colonies from which they were derived, preventing them from differentiating [71, 72]. Under the defined culture conditions, primitive endoderm-like cells, constitutively emerged from their clonally-related precursors, acted in a paracrine fashion to supports efficient clonal expansion of single pluripotent hESCs through the activinA-SMAD pathway and mediated by ZNF206 [71, 72]. Such defined conditions derived their efficacy from enabling the spontaneous unfolding of inherent early embryogenesis processes *in vitro* that emulated the *in vivo* maintenance of the pluripotent epiblast developed from the ICM. Undifferentiated hESCs maintained under these defined biologics-free (i.e., animal-free, exogenous feeder-, serum-, and conditioned-medium-free) conditions could be clonally expanded for prolonged periods in culture (with either trypsin or mechanical dissociation) and form teratomas (containing derivatives of all three embryonic germ layers after grafting into Severe Combined Immuno-Deficient [SCID] mice), suggesting that they remained pluripotent and self-renewing [52, 71, 72]. Our inventions allow all poorly-characterized and unspecified biological additives, components, and substrates in the culture system (including those derived from animals) removed, substituted, or optimized with defined human alternatives [52, 71, 72]. Such a system emulating human embryonic development then served as a platform for well-controlled efficient derivation of clinically-relevant specialized human cells, as illustrated by directed cardiac and neural differentiation Figs. (**5, 6**), from pluripotent hESCs by the simple provision of small molecules, serving as prototypical cell type fates for clinical translation [52]. 

## MANIPULATION OF hESCs

Genetic modifications of hESCs have been used to improve assay, quantification, imaging, purification, and genomic analysis of hESCs, which have resulted in patents on various reporter constructs and genetically altered hESC lines for stem cell pluripotence and differentiation analysis as well as the systems and methods of introducing exogenous or foreign genetic materials into human stem cells, including viral DNA vectors, bacterial artificial chromosome (BAC) reporters, and transposons, to make such modified cell lines [73-80]. Identification of pluripotence-associated molecules and markers, including genes, cDNAs, micro-RNA, and proteins, would allow manipulation of hESCs at multiple regulatory pathways and levels for research and biotechnology purposes [81-83]. The genetically-engineered hESCs have been used to facilitate biological studies, therapeutic development, and drug screening processes [84, 85]. Several improved manipulation methods for culturing and monitoring hESCs, such as eliminating spontaneously differentiated cells, cryoperserving pluripotent human cells, and imaging living cells on a cell culture substrate have also been claimed and granted patents by the USPTO [86-88].

## DIFFERENTIATION OF hESCs

Although undifferentiated hESCs have been claimed utilities in treating cancer by secreting factors to prevent tumor formation and progression and in controlling the immune system for minimizing the alloreactivity of tissue transplants [89, 90], the major driving force of hESCs is their property of differentiating into any cell type in the human body that has imminent therapeutic utility for human tissue and organ regeneration. Pluripotent hESCs have the unconstrained capacity for long-term stable undifferentiated growth in culture and the theoretical potential for differentiation into all somatic cell types. Therefore, they have been regarded as an ideal source to provide an unlimited supply of large-scale well-characterized human multipotent somatic stem/progenitor/precursor cells and specialized mature cells for cell replacement or regeneration therapies in treating a wide range of diseases and injuries. The major challenge in using pluripotent cells for cell-based therapy is to produce a large and uniform population of lineage-committed transplantable cells *in vitro*. In addition, *in vivo* studies using animal models of disease will be critical to examine the physiological functions of engrafted hESC derivatives, evaluate their efficacy in repair, and more importantly, affirm their safety, such as a lack of teratoma or inappropriate cell type formation. The hESCs have the multi-lineage inclination of pluripotent cells [1]. The hESCs can differentiate spontaneously* in vitro* into cells of all germ layers by forming embryoid bodies, which are multi-lineage aggregates in suspension [91-93]. Embryoid bodies contain mixed populations of cells originated from endoderm, mesoderm, and ectoderm that are differentiated to variable degrees as well as undifferentiated and partially differentiated hESCs. Only a small fraction of cells in the embryoid bodies pursues a given lineage. In addition, the simultaneous emergence of substantial widely divergent undesired cell types that may reside in all three embryonic germ-layers in the embryoid bodies makes directing hESC differentiation *en a particular route* unpredictable and unreliable. Therefore, methods such as using apoptotic gene or selective antibody to deplete undifferentiated hESCs, co-culturing with mouse stromal cells, using cytokines to induce floating embryoid body have been used to enrich the desired populations and improve homogeneity of desired cell types [94-99]. One particularly successful case is the claims that treating embryoid bodies with butyrate and a mixture of cytokines or hormones yields a remarkably high proportion of cells with phenotypic characteristics of liver cells [100-103]. To date, neural, pancreatic, and cardiac lineages have been the most claimed differentiation areas of hESCs as a result of intense interest in developing cell-based therapies for neurodegenerative diseases, paralysis, diabetes, and heart diseases Tables **3**-**5**. In addition, pluripotent hESCs have been claimed differentiation into many other clinically-relevant lineages and cell types *in vitro* through multi-lineage germ-layer induction, including hematopoieticcells [96, 97], hepatocytes [100-103], connective tissue progenitors [104], osteoblast and chondrocyte precursors [105-107], and mesenchymal stem cells [108, 109]. However, developing practical and efficient technology to be able to produce a large population of uniform uncontaminated progenies from hESCs for therapeutic application remains as a major challenge.

### Neural Differentiation of hESCs

There is a large unfulfilled need for a clinically-suitable human neuronal cell source for repair or regeneration of the damaged central nervous system (CNS) structure and circuitry in today's healthcare industry. Cell-based therapies hold great promise to restore the lost nerve tissue and function for CNS disorders. However, cell therapies based on fetal or adult CNS-derived human neural stem cells (hNSCs) have encountered supply restriction and difficulty to use in the clinical setting due to their limited expansion ability in culture and failing plasticity after extensive passaging [2, 4, 5]. Despite some beneficial outcomes, the CNS-derived hNSCs appear to exert their therapeutic effects primarily by their non-neuronal progenies through producing trophic and neuroprotective molecules to rescue the endogenous cells, rather than replacement or reconstruction of the damaged CNS structure and circuitry [2, 4, 5]. Alternatively, the genetically stable hESCs proffer cures for a wide range of neurological disorders by supplying the diversity of human neuronal cell types in the developing CNS for regeneration. Substantial neural differentiation appears to occur at relatively early stages in hESC cultivation under conditions that induce differentiation [110-120] Table **3**, consistent with the early development of the CNS from the cells of ICM or epiblast in embryogenesis. Parkinson’s Disease (PD), a neuronal degenerative disorder associated with a loss of midbrain neurons that synthesize the neurotransmitter dopamine (DA), has long been regarded as an ideal model for testing the safety and efficacy of various therapeutic strategies against diseases of the CNS [5]. Therefore, generating dopaminergic neurons from hESCs has been an interesting development. To date, most claims are on Nestin positive neural progenitor or precursor cells (NPCs), enriched or selected from embryoid bodies, that can differentiate into heterogeneous populations of neurons, including dopaminergic neurons, and glial cells [110, 111, 114-120] Table **3**. Developing strategies to generate a large and uniform population of lineage-committed transplantable cells *in vitro* is vital to using pluripotent cells for cell-based therapy. Homogenous GalC positive oligodendrocyte progenitor populations predifferentiated from hESCs appear to be able to survive and differentiate into oligodendrocytes, following transplantation, to enhance remyelination and improve motor function following acute (7 days after injury), but not chronic (10 months) injury in rodents [121] Table **3**. Generation of a homogenous population of hESC-derived oligodendrocyte progenitor cells, GRNOPC1, allows Geron Corporation to obtain the US Food and Drug Administration (FDA) approval for first clinical trial of hESC-derived cell products in patients with a complete spinal cord injury within 14 days of the injury, which began in October, 2010 [121-123]. The launch of first clinical trial of hESC therapy is quickly followed by FDA approval for two more clinical trials to treat Stargardt's macular dystrophy and age-related macular degeneration after Advanced Cell Technology has established methods for producing an enriched population of human retinal pigment epithelium (RPE) cells or their progenitors that may lead to improved cell-based therapies for retinal degeneration [124, 125] Table **3**.

Although neural lineages appear at a relatively early stage in hESC differentiation, treating hESC-differentiated embryoid bodies with retinoic acid only slightly increased the low yield of neurons [111, 113]. Retinoic acid was not sufficient to induce the neuronal differentiation of hESCs maintained on feeder cells [52]. However, small molecule retinoic acid was rendered sufficient to induce hESCs maintained in the defined culture system to transition from pluripotence exclusively to a neuroectodermal phenotype [52]. Upon exposure of undifferentiated hESCs to retinoic acid under the defined culture, all the cells within the colony underwent morphology changes to large differentiated cells that ceased expressing pluripotence-associated markers, as indicated by Oct-4, and began expressing neuroectoderm-associated markers, such as HNK1 and AP2, but not Nestin, consistent with a neuroectoderm phenotype Fig. (**5A**). These differentiated cells continued to multiply and the colonies increased in size, proceeding spontaneously to express the early neuronal marker β-III-tubulin, but not markers associated with other lineages. The more mature neuronal marker Map-2 began to appear in areas of the colonies where cells had piled up Fig. (**5A**). The neural-induced hESCs were detached and then allowed to form floating cellular clusters in a suspension culture to continue the neural differentiation process. These differentiating floating cellular clusters became almost uniformly positive for β-III-tubulin, therefore termed “neuroblasts” Fig. (**5B**). Upon removal of bFGF and after permitting the neuroblasts to attach, β-III-tubulin- and Map-2-expressing, neurite-bearing cells and pigmented cells (typical of those in the ventral mesencephalon) began to appear with high efficiency Fig. (**5C**). Nurr1, a member of the orphan nuclear hormone receptor super-family, has been implicated in neuronal development, particularly ventral mesencephalic development and activation of the tyrosine hydroxylase gene, the rate-limiting step in catecholaminergic and dopaminergic neuronal differentiation [5]. Interestingly, in undifferentiated hESCs, Nurr1 localizes to the cell-surface and cytoplasm, consistent with its being inactive Fig. (**5D**). However, upon exposure of the hESCs to retinoic acid, Nurr1 translocated to the nucleus, coincident with the appearance of the neuroectodermal cells, and continued to assume its strong expression and nuclear localization at the later neuronal stages Fig. (**5D**). Accordingly, a large proportion of these hESC-derived neuronal cells began to express tyrosine hydroxylase Fig. (**5D**), consistent with the early stages of acquiring catecholaminergic or dopaminergic potential. Similarly, a proportion of Map-2**+** cells began to express Hb9 and Lim3, markers implicated in the early stages of motor neuron development, another ventrally-located neuronal population Fig. (**5D**). Sonic hedgehog (Shh) appeared to promote the proliferation of those ventral neuronal cells Fig. (**5D**).

### Cardiac Differentiation of hESCs

Cardiovascular disease is a major health problem and the leading cause of death in the Western World. In the United States, around 5 millions survive heart failure but live with insufficient cardiac function, and about 550,000 new cases are diagnosed annually. Heart attacks, known as myocardial infarction, are the main cause of death in patients with cardiovascular disease. Around 1/3 of the patients suffering from heart attacks each year die suddenly before reaching the hospital. In the remaining patients who survive their initial acute event, the damage sustained by the heart may eventually develop into heart failure, with an estimated median survival of 1.7 years in men and 3.2 years in women [126, 127]. To date, the lack of a suitable human cardiac cell source has been the major setback for regenerating the human myocardium, either by cell-based transplantation or by cardiac tissue engineering [126, 127]. Cardiomyocytes become terminally-differentiated soon after birth and lose their ability to proliferate. Damaged and diseased cardiomyocytes are removed largely by macrophages and replaced by scar tissue. There is no evidence that stem/progenitor cells derived from other sources, such as the bone marrow or the cord blood, are able to give rise to the contractile heart muscle cells following transplantation into the heart [126, 127]. The need to regenerate or repair the damaged heart muscle has not been met by adult stem cell therapy, either endogenous or via cell delivery. Due to the prevalence of cardiovascular disease worldwide and acute shortage of donor organs, there is intense interest in developing hESC-based therapies as an alternative approach. The heart is the first organ formed from the cells of the ICM or epiblast of the blastocyst in early embryogenesis. The blastocyst-derived hESCs can differentiate spontaneously* in vitro* into cardiomyocytes that display functional cardiomyocyte phenotype after a prolonged period in culture by going through a multi-lineage aggregate stage, but do so with < 2% efficiency [128, 129] Table **4**. Therefore, various cardiomyocyte differentiation inducing factors, such as Activin-A, BMP-4, BMP antagonists, and exogenous cells, have been used to treat hESC aggregates, either in suspension or adherent, which appear slightly improve the yield of cardio-myocytes [130-139] Table **4**. In contrast, vascular progenitor cells, smooth muscle cells, and endothelial cells can be generated from hESCs under differentiation conditions that prevent cell aggregation with abundances [140-143]. 

Developing strategies to channel the wide differentiation potential of pluripotent hESCs exclusively and predictably to a desired cardiac phenotype is critical for their therapeutic utility. The development of a defined platform for the maintenance of hESCs renders nicotinamide sufficient to induce a cascade of cardiac differentiation events directly from pluripotent hESCs by promoting the expression of the earliest cardiac marker Csx/Nkx2.5 and triggering cardiac specification and progression to beating cardiomyocytes efficiently Fig. (**6**) [52]. Nicotinamide was not sufficient to induce the cardiac differentiation of hESCs maintained on feeder cells or through embryoid bodies [52]. Upon exposure of hESCs to nicotinamide, all the cells within the colony underwent morphology changes to large differentiated cells that down-regulated the expression of Oct-4 and began to express the cardiac specific transcription factor (Csx) Nkx2.5, but not markers associated with other lineages, consistent with a cardiomesoderm phenotype Fig. (**6A**). Increased intensity of Nkx2.5 was usually observed in areas of the colonies where cells began to pile up Fig. (**6A**). The cardiac-induced hESCs were detached and then allowed to form floating cellular clusters in a suspension culture to continue the cardiac differentiation process. These differentiating floating cellular clusters relatively uniformly expressed Nkx2.5 in suspension, therefore termed “cardioblasts” Fig. (**5B**). After permitting the cardioblasts to attach, beating cardiomyocytes began to appear with a drastic increase in efficiency Fig. (**6B**). 

### Pancreatic Differentiation of hESCs

The hope to produce insulin-secreting cells from hESCs for treating diabetes currently affecting 250 million people worldwide has intensified the competition on developing such techniques. Autoimmune Type 1 diabetes, also known as juvenile-onset diabetes, is caused by a person's own immune system mistakenly destroying their insulin-producing cells in the pancreas, known as beta cells, which produce insulin in response to food intake to control the level of blood sugar. One of the major challenges in generating beta cells from hESCs is to determine the necessary molecular and cellular cues that direct efficient and predicable pancreatic differentiation of hESCs. The normal developmental pathways that generate pancreas in embryogenesis remain poorly understood. As a result, directing hESCs along specific pathways of pancreatic differentiation in a systematic manner has proved difficult. So far, several groups have developed methods to generate cells expressing endoderm and pancreatic endoderm markers enriched from hESCs [144-153] Table **5**. However, whether these hESC-derived early stage pancreatic cells can produce insulin in response to glucose stimulation *in vivo* remain to be shown.

## CURRENT & FUTURE DEVELOPMENTS

The pluripotence of hESCs implies such cells’ tremendous potential for tissue and function restoration. However, how to channel the wide differentiation potential of human pluripotent cells efficiently and predictably to a desired phenotype has been a major challenge for both developmental study and clinical translation of their therapeutic potential. Conventional approaches rely on multi-lineage differentiation of pluripotent cells through germ-layer induction, which yields mixed populations of cell types that may reside in three embryonic germ layers and often makes desired differentiation not only inefficient, but uncontrollable and unreliable as well [2]. Following transplantation, these hESC-derived grafts tend to display not only a low efficiency in generating the desired cell types necessary for reconstruction of the damaged structure, but also phenotypic heterogeneity and instability, hence, a high risk of tumorigenicity [2, 9, 154]. In view of growing interest in the use of human pluripotent cells, teratoma formation and the emergence of inappropriate cell types have become a constant concern following transplantation [2, 9, 154]. Developing more practical approaches that permit to channel the wide differentiation potential of pluripotent hESCs efficiently and predictably to a desired phenotype is vital to harnessing the power of hESC biology for safe and effective clinical translation. Resolving the essential components for sustaining pluripotence of hESCs in a defined culture system has served as a platform for *de novo* derivation of clinically-suitable hESCs that can be directly induced into large supplies of neural- or cardiac-committed progenies across the spectrum of developmental stages for safe and effective clinical translation [52]. Defined conditions for induction of cardioblasts and neuroblasts direct from pluripotent hESCs enable using small molecules for well-controlled efficient derivation of an unlimited supply of human cardiac and neuronal cells across the spectrum of developmental stages from hESCs for cell-based therapeutics [52]. Such development will open a new dimension of small molecule-mediated direct control and modulation of hESC fate, including using signal molecules and microRNAs, when deriving an unlimited supply of clinically-relevant lineages from pluripotent hESCs for regenerative therapies.

Standard stem cell differentiation protocols involve cultivation in 2-dimensional (2D) settings, whereas *in vivo* organogenesis requires a 3D setting to provide the spatial and temporal controls of cell differentiation necessary for the formation of functional tissues [155, 156]. The traditional methods of 2D culture often result in unpredictable stem cell function and behavior *in vivo* following transplantation [155, 156]. Developing strategies of complex 3D models of human embryogenesis and organogenesis will provide a powerful tool that enables more rigorous experimentation under conditions that are tightly regulated and authentically representing the *in vivo* spatial and temporal patterns [37]. It will go beyond “flat biology” to increase the biological complexity of human-based *in vitro* models and assays to mimic the *in vivo* human organ systems and functions, which are controllable, reproducible, and scalable, and can be monitored and validated against responses on multiple hierarchical levels. The development and utilization of the 3D human embryonic model will facilitate rapid progress in the identification of molecular and genetic therapeutic targets for prevention and treatment of diseases. It will lead to reconstituting fully competent human tissues and organs in 3D from hESCs to meet the medical need of replacement organs for transplantation therapies. Although ethical debate about the patentability of hESCs and policy makers on Funding issues are still lagging behind the scientific developments, in the case of many life-threatening diseases, supporting such research is crucial to driving the advance of medicine to provide optimal regeneration and reconstruction treatment options for the damaged or lost functional tissues and organs that have been lacking. Future patent filings on breakthroughs of hESC research will enable clinical practices to improve the function, wellness, and overall quality of life.

## Figures and Tables

**Fig. (1):**
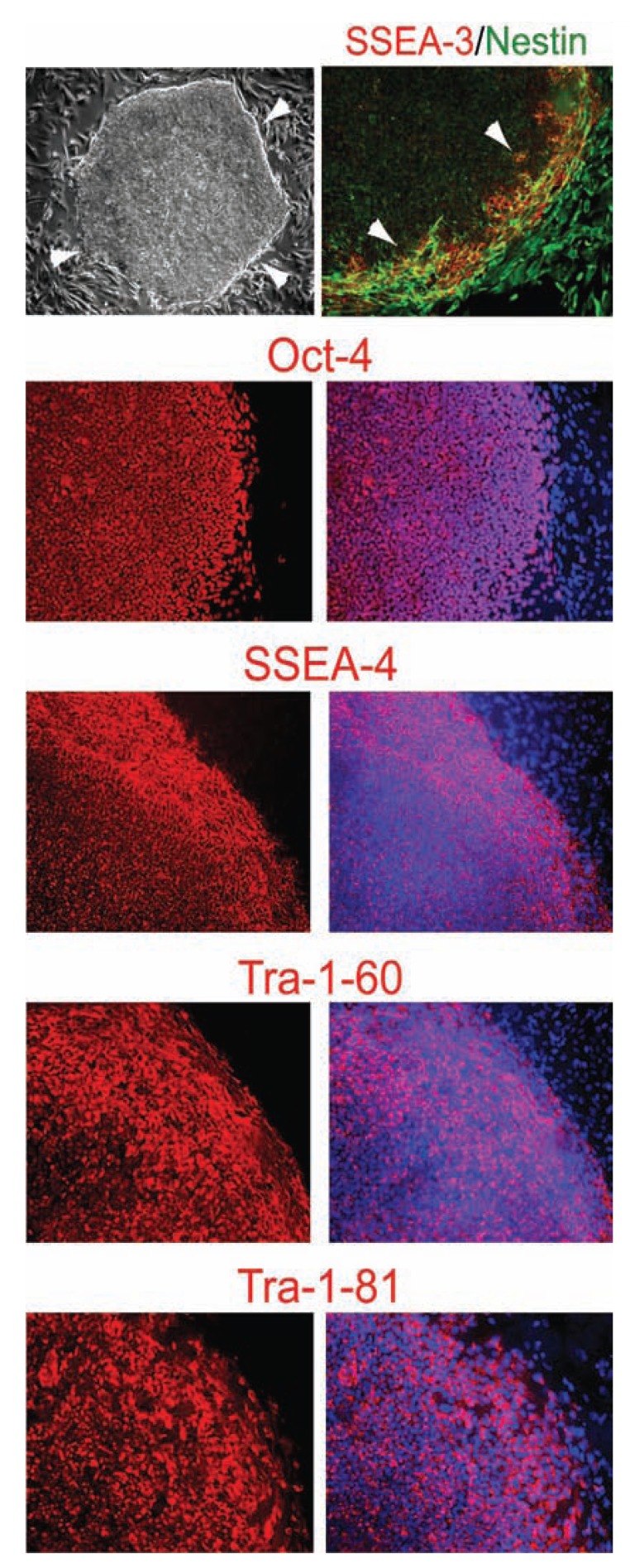
bFGF is a critical component that sustains undifferentiated growth of hESCs on human feeders. Phase contrast image shows the highly compact undifferentiated morphology of a hESC colony on human feeder cells. White arrows delineate the edge of a hESC colony. Immunofluorescence images show that hESCs inside the colonies express the undifferentiated hESC markers Oct-4 (red), SSEA-4 (red), Tra-1-60 (red), and Tra-1-81 (red). Cells at the edge of the colonies exhibit the classic flattened epithelial morphology indicative of the onset of differentiation, and express SSEA-3 (red) and Nestin (green). Cells that have migrated outside the colonies continued to differentiate into large elliptoid-appearing cells that persist in expressing Nestin, but cease expressing SSEA-3, Oct-4, SSEA-4, Tra-1-60, and Tra-1-81. All cells are revealed by DAPI staining of their nuclei (blue) in the merged images.

**Fig. (2):**
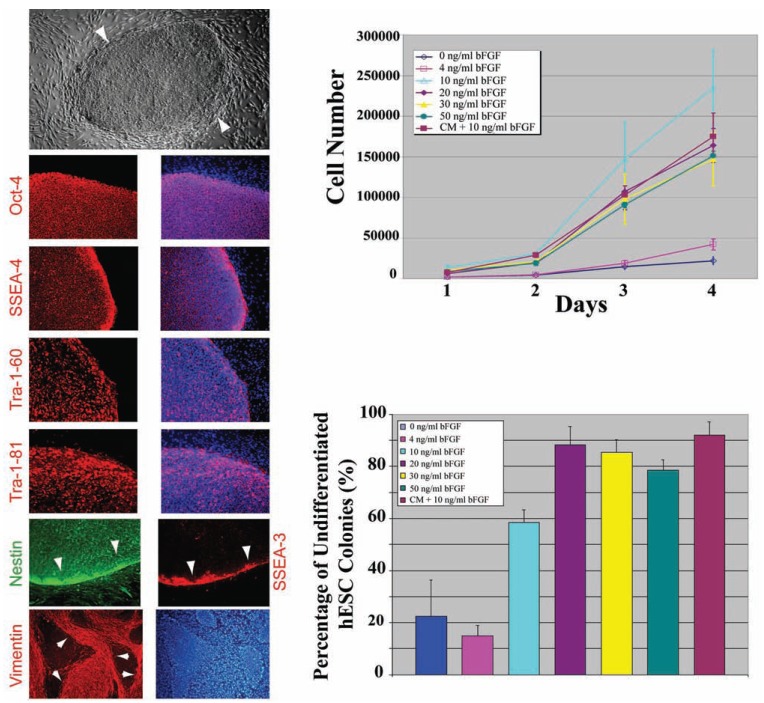
bFGF is a critical component that sustains undifferentiated growth of hESCs on laminin/collagen (Matrigel). Phase contrast images show the highly compact undifferentiated morphology of a hESC colony on laminin/collagen. White arrows delineate the edge of a hESC colony. Immunofluorescence images show that hESCs inside the colonies express the undifferentiated hESC markers Oct-4 (red), SSEA-4 (red), Tra-1-60 (red), and Tra-1-81 (red). Cells at the edge of the colonies exhibit the classic flattened epithelial morphology indicative of the onset of differentiation, and express SSEA-3 (red) and Nestin (green). Cells that have migrated outside the colonies continued to differentiate into large elliptoid-appearing cells that persist in expressing Nestin and Vimentin (red), but cease expressing SSEA-3, Oct-4, SSEA-4, Tra-1-60, and Tra-1-81. All cells are revealed by DAPI staining of their nuclei (blue) in the merged images. bFGF short-term proliferation assay shows that, without bFGF or with a low concentration of bFGF (4 ng/ml), hESCs displayed significantly slow growth rates. With bFGF at a concentration ranging from 10 to 50ng/ml, hESCs displayed a comparable growth rate as those maintained in MEF-conditioned media (CM). bFGF dose-response assay shows that hESCs maintained in media containing 20 ng/ml bFGF exhibited the highest undifferentiated percentage.

**Fig. (3):**
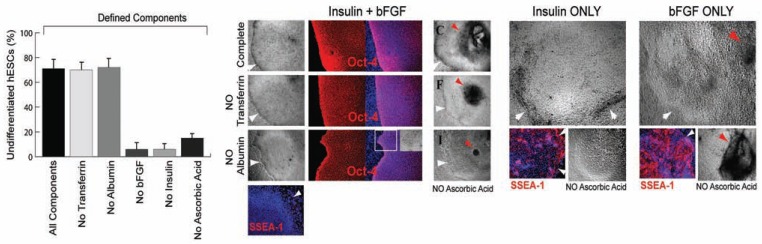
bFGF, insulin, ascorbic acid, and laminin are minimal essential requirements for the maintenance of undifferentiated hESCs. Quantitative analysis of defined components with hESCs seeded on purified human laminin and cultivated in a base medium indicates that bFGF, insulin, and ascorbic acid are essential components for maintaining substantial numbers of hESCs in undifferentiated state, indicated by Oct-4 positive. With all the components or in the absence of transferrin, a majority of hESC colonies displayed a highly compact undifferentiated morphology and expressed Oct-4 (red). In the absence of albumin, hESC colonies were more flat and spread out (white square delineates the same area shown in the inset), but a large proportion continued to express Oct-4 and exhibited a highly compact morphology. However, if ascorbic acid was omitted from the media (NO Ascorbic Acid), the colonies often became very dense in the center and cyst-like (red arrows). Undifferentiated hESCs maintained in media containing both bFGF and insulin do not express SSEA-1 (red). Absence of either bFGF or insulin induces complete differentiation, as indicated by SSEA-1 expression. Large round cells were usually present in media that contained only insulin, and elliptically-shaped cells were present in media that contained only bFGF. Absence of ascorbic acid (NO Ascorbic Acid) resulted in slower cell growth in media containing only insulin, but accelerated the differentiated growth in the dense centers of colonies in media containing only bFGF (red arrows). White arrows delineate the edge of hESC colonies. All cells are indicated by DAPI staining of their nuclei (blue).

**Fig. (4):**
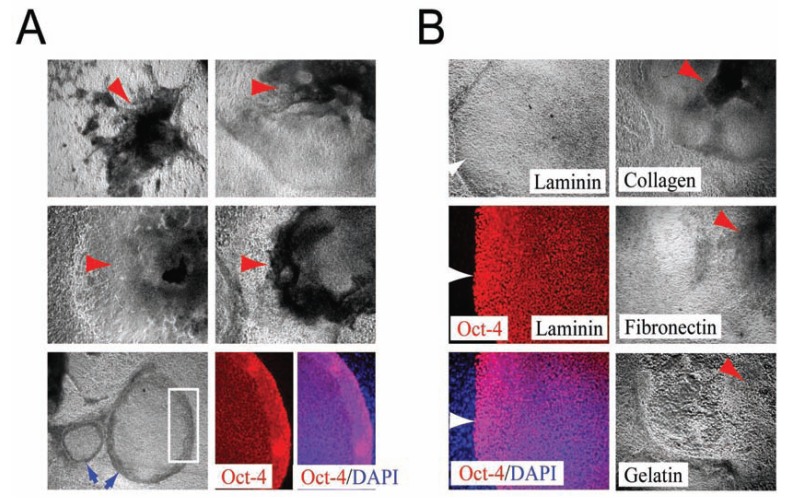
(**A**) Other growth factors can not replace bFGF for the maintenance of undifferentiated hESCs. hESC colonies maintained in aFGF, EGF, IGF-I, IGF-II, PDGF, VEGF, activin-A, and BMP-2 generally display a more differentiated morphology that consists of dense centers containing cyst-like structures and cells heaping upon each other (red arrows). Note that, although most cells are differentiated, a minority of the small colonies (<30%) retain a compact morphology (blue arrows) and continue to express Oct-4 (red). White square indicates the approximate area that is visualized at higher magnification in the right. DAPI staining is blue. (**B**) Determining the minimal essential matrix. hESCs maintained on laminin have a classic undifferentiated morphology and express Oct-4 (red). DAPI staining of their nuclei is blue. White arrows delineate the edge of a hESC colony. In contrast, hESC colonies maintained on fibronectin, collagen IV, or gelatin displayed a more differentiated morphology within their first passage.

**Fig. (5):**
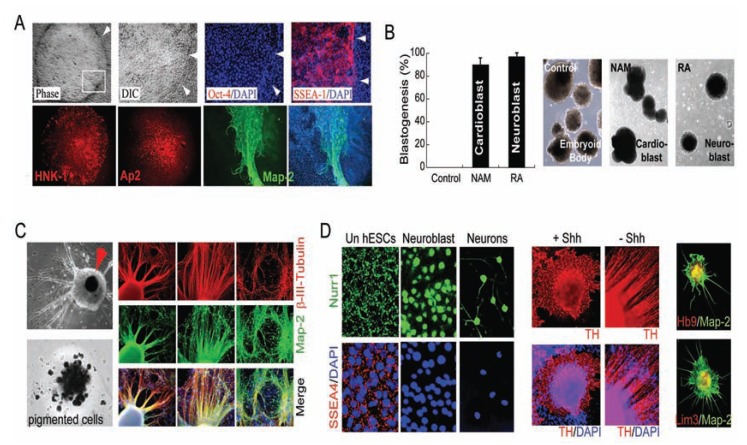
Retinoic acid (RA) signals neural induction direct of pluripotence under defined conditions. (**A**) Upon exposure of hESCs to RA under the defined culture system, large differentiated Oct-4 (red) negative cells within the colony began to emerge. RA-induced differentiated Oct-4-negative cells began to express SSEA-1 (red), HNK1 (red), and AP2 (red), consistent with early neuroectodermal differentiation. These cells continued to mature ultimately expressing the neuronal marker Map-2 (green), usually in areas where cells began to pile up. All cells are indicated by DAPI staining of their nuclei (blue). White arrows delineate the edge of a hESC colony. (**B**) The induced hESCs formed cardioblasts (Nkx2.5+, with nicotinamide [NAM], see Fig. **6**) or neuroblasts (β-III-tubulin+, with RA) in suspension, as compared to germlayer-induced embryoid bodies (EBs) derived from hESCs without treatment (Control). (**C**) RA treatment induces differentiation towards a neuronal lineage with a drastic increase in efficiency, as assessed by the percentages of cells that expressed β-III-tubulin (red) and coexpressed Map-2 (green) (arrow, pigmented cells). (**D**) Nurr1 translocates to the nucleus upon exposure of hESCs to RA. A large subpopulation of these hESC-derived neuronal cells progressed to express tyrosine hydroxylase (TH, red) in the presence sonic hedgehog (+Shh) or absence Shh (–Shh). A subpopulation of these hESC-derived Map-2-positive (green) cells began to express Hb9 (red) and Lim3 (red) (shown in a 3D matrix). All cells are indicated by DAPI nuclear staining (blue).

**Fig. (6):**
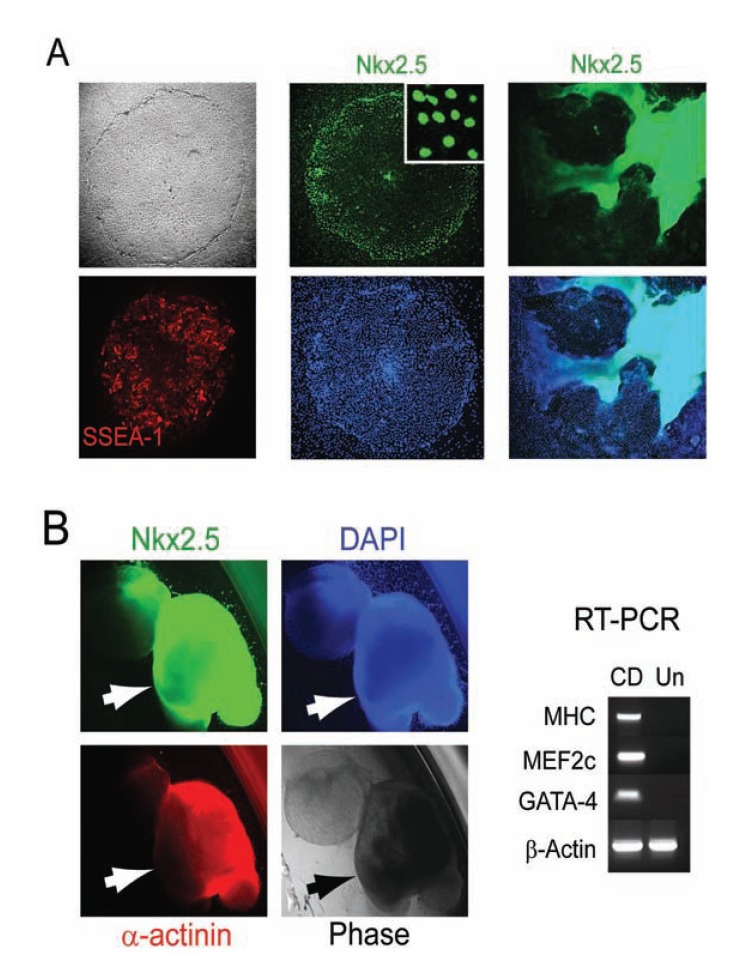
Nicotinamide (NAM) signals cardiac induction direct of pluripotence under defined conditions. (**A**) Upon exposure of hESCs to NAM under the defined culture system, large differentiated Oct-4 (red) negative cells within the colony began to emerge. NAM-induced Oct- 4-negative cells began to express SSEA-1 (red), Nkx2.5 (green), consistent with early cardiac differentiation. Progressively increased intensity of Nkx2.5 was usually observed in areas of the colony where cells began to pile up. (**B**) NAM-induced hESCs yielded beating cardiomyocytes with a drastic increase in efficiency, as assessed by the percentages of cellular clusters that displayed rhythmic contractions (arrows), and immunopositive for Nkx2.5 (green) and α-actinin (red), and expressed cardiomyocyte markers cardiac MHC, MEF2c, and GATA-4 (CD), as compared to undifferentiated hESCs as the control (Un). All cells are indicated by DAPI nuclear staining (blue).

**Table 1:** Patents Related to Derivation of Pluripotent Human Embryonic Stem Cells.

Publication Number and Date	Title and Ref. No.	Inventors	Division or Continuation of Primate/Animal Patent	Feeder	Marker, Growth Condition, or Method	Isolation Stage of Embryo	Karyotype	Covered Differentiation
US7781216 (**2010**)	Spontaneous differentiation of hESCs in culture [28]	Thomson JA	US5843780 (**1998**)*, US6200806 (**2001**)*, US7029913 (**2006**)*	Mouse embryonic fibroblasts	SSEA-1(-)	ICM of human blastocyst	Normal euploid	Endoderm, mesoderm, ectoderm
US6875607 (**2005**)	ESCs [29]	Reubinoff BE, *et al.*		Mouse or human fibroblasts		ICM of human blastocyst		Committed progenitor cells, mature somatic cells
US7153684 (**2006**)	Pluripotential ESCs and methods of making same [30]	Hogan BLM	US5690926 (**1996**)	Feeder cells or extracell-ular matrix	SSEA-1(+), alkaline phosphatase(+)		Normal euploid	Embryoid bodies, multiple differentiated cells
US7294508 (**2007**)	Isolation of ICM for the establishment of hESC lines [31]	Parikh FR, *et al.*			Laser ablation	ICM of human blastocyst		
US7811817 (**2010**)	Establishment of a hESC line using mammalian cells [32]	Mandal A, *et al.*			SSEA-1 (-), SSEA-4(+); TRA-1-60 (+); TRA-1-81 (+); alkaline phosphatase (+), Oct-4 (+)	ICM of human blastocyst	Normal euploid	Endoderm, mesoderm, ectoderm
US6921632 (**2005**)	HESCs derived from frozen-thawed embryo [33]	Lim JH, *et al.*		Mouse embryonic fibroblast STO cells		ICM of Cryo-preserved human blastocyst embryo		
US2009104697 (**2009**)	Method of differentiation of morula or ICM cells and method of making lineage-defective ESCs [34, 35]	Cibelli J, *et al.*				Human morula or ICM of human blastocyst		Progenitor cells
WO2006036164 (**2006**)	Morula derived ESCs [36]	Strelchenko N, Verlinsky Y		Feeder cells		Human morula		
US2010093091 (**2010**)	Undifferentiated stem cell culture systems [37, 38]	Reubinoff B, Steiner D		Free of feeder cells or micro-carriers	Suspension culture conditions	ICM of human blastocyst		Endoderm, mesoderm, ectoderm
US7442548 (**2008**)	Culturing hESCs in medium containing pipecholic acid and gamma amino butyric acid [42, 43]	Thomson JA, Ludwig T		Free of feeder cells or conditioned medium	FGF at least 40 ng/ml and a matrix of human proteins selected from collagen, fibronectin, vitronectin, and laminin	ICM of human blastocyst	Normal euploid	

* under USPTO reexamination.

**Table 2:** Patents Related to Culture Techniques of Human Embryonic Stem Cells and Pluripotence Maintenance.

Publication Number and Date	Title and Ref. No.	Inventors	Division or Conti-nuation of Patent	Covered Claims
US6642048 (**2003**)	Conditioned media for propagating hPSCs [39, 44]	Xu C, Gold JD		Conditioned media by mouse or human embryonic mesenchymal and fibroblast-like cell lines to support proliferation of pluripotent hESCs while inhibiting differentiation in an environment free of feeder cells.
US7217569 (**2007**)	Clonal cultures of primate ESCs [45, 46]	Thomson JA	US7005252 (**2006**)	Culturing primate ESCs on a prolonged and stable basis in the presence of exogenously supplied FGF at least 4 ng/ml and a mouse embryonic fibroblast feeder layer, and in the absence of animal serum.
US7790455 (**2010**)	Human foreskin fibroblast conditioned media for culturing ESCs [47, 48]	Amit M, Itskovitz-Eldor J	US7267981 (**2007**)	A cell culture comprising human foreskin cells, or human foreskin cell-conditioned media capable of maintaining stem cells in an undifferentiated state.
US7682826 (**2010**)	HESCs and culturing methods thereof [49]	Yang MJ, *et al.*		Maintaining the undifferentiated growth of hESCs in an extracellular matrix isolated from inactivated feeder cells or a conditioned medium by inactivated feeder cells selected from the group consisting of human foreskin fibroblasts and primary mouse embryonic fibroblasts.
US7432104 (**2008**)	Methods for the culture of hESCs on human feeder cells [50]	Mitalipova M, Lyons I		Culturing pluripotent hESCs with human granulosa feeder cells or their conditioned media, muscle cells, fallopian ductal epithelial cells, bone marrow stromal cells, and skin fibroblasts.
US2004253721 (**2004**)	Methods of derivation and propagation of undifferentiated hESCs on feeder-free matrices and human feeder layers [40, 51]	Bongso A, *et al.*		Derivation and propagation of undifferentiated hESCs on human feeder layers including human fetal muscle, human fetal skin, human adult fallopian tube fibroblasts and human adult skin cells, and/or in the absence of a feeder layer.
US7592175 (**2009**)	Methods of preparing feeder cells-free, xeno-free hESCs and cell cultures prepared using same [53]	Amit M, Itskovitz-Eldor J		Culturing hESCs on a fibronectin matrix and a tissue culture medium which comprises TGFβ, bFGF, LIF, and free of xeno- and feeder cells contaminants.
US7413902 (**2008**)	Feeder-free culture method for ESCs or primate primordial stem cells [54, 55]	Bodnar AG, *et al.*	US6800480 (**2004**)	The basic medium combined with a nutrient serum and a substrate of an extracellular matrix component derived from fibroblasts effective to support the growth of hESCs.
US7455983 (**2008**)	Medium for growing hESCs [56-58]	Xu C, *et al.*	US7297539 (**2007**), US7410798 (**2008**)	Culturing hESCs in the presence of an extracellular matrix in a medium that comprises a FGF at a concentration of at least 40 ng/ml and Flt-3 ligand at a concentration of 15 ng/ml, and free of feeder cells.
US7439064 (**2008**)	Cultivation of hESCs in the absence of feeder cells or without conditioned medium [59]	Thomson JA, Levenstein M		Culturing hESCs in an environment essentially free of mammalian fetal serum, without the need for feeder cells or for exposure of the medium to feeder cells, and in a medium including amino acids, vitamins, salts, minerals, transferring, insulin, albumin, and a FGF at least 100 ng/ml.
US7449334 (**2008**)	Medium containing pipecholic acid and gamma amino butyric acid and culture of ESCs [60]	Thomson JA, Ludwig T		Culturing hESCs in high levels of FGF (at least 40 ng/ml), gamma amino butyric acid, pipecholic acid, lithium, TGFβ and a matrix of human proteins that comprises at least three of the proteins selected from collagen, fibronectin, vitronectin, and laminin without feeder cells, conditioned medium, or animal products.
US7514260 (**2009**)	Feeder independent extended culture of ESCs [61, 62]	Xu RH, Thomson JA		Culturing hESCs in an antagonist of BMP and FGF without feeder cells or conditioned medium.
US2009191634 (**2009**)	Methacrylate surfaces for cell culture, methods of making and using the surfaces [64, 66]	Martin AW, *et al.*		A synthetic cell culture surface prepared from a polymerized blend of at least two (meth)acrylate monomers which supports the growth of undifferentiated hESCs in defined media augmented with FBS.
US2008241919 (**2008**)	Defined media for pluripotent stem cell culture [52]	Parsons XH, Snyder EY	US2005233446 (**2005**), US2007010011 (**2007**)	Formulation of minimal essential defined components for maintaining the long-term stable growth of undifferentiated hESCs, including bFGF (20 ng/ml), insulin, ascorbic acid, and laminin, for the derivation and large-scale production of pluripotent hESCs in optimal yet well-defined biologics-free culture conditions from which they can be efficiently directed towards a lineage-specific differentiated fate, illustrated by cardiac or neuronal differentiation, in connection with clinical applications and in drug discovery processes.

**Table 3:** Patents Related to Neural Differentiation of Human Embryonic Stem Cells.

Publication Number and Date	Title and Ref. No.	Inventors	Division or Continuation of Patent	Initiating Differentiation	Differentiating Agents or Methods	Covered Human Neural Cells	Neural Markers or Phenotypes
US7763463 (**2010**)	Use of cyclic AMP and ascorbic acid to produce dopami-nergic neurons from ESCs [110, 111]	Carpenter MK, Thies RS	US6833269 (**2004**)	Embryoid bodies	Neurotrophins, cAMP, ascorbic acid; noggin, follistatin; retinoic acid, sonic hedgehog, isolation	NPCs, or neurons and glial cells, or dopaminergic neurons	A2B5, PSNCAM, MAP-2, Nestin (>60%), or tyrosine hydroxylase (>5%)
US7560281 (**2009**)	Use of TGF beta superfamily antagonists to make dopaminergic neurons from ESCs [112]	Carpenter MK, Thies RS		Monolayer culture on a solid surface without extracellular matrix	Noggin, follistatin, neurotrophin, isolation	Dopaminergic neurons	Tyrosine hydroxylase
US7045353 (**2006**)	Directed differentiation of hESCs [113]	Benvenisty N		Embryoid bodies	Nerve growth factor, retinoic acid	Neuronal cells	
US7674620 (**2010**)	Derivation of terminally differentiated dopaminergic neurons from hESCs [114]	Totey SM, Ravindra G		Enrich NPCs by NCAM sorting	N-acetyl cysteine, TGF-beta3, interleukin1beta, isolation	NPCs, or dopaminergic neurons (>60%) & serotonergic neurons (>30%)	Nestin, NCAM, or tyrosine hydroxylase
US6887706 (**2005**)	Method of *in vitro* differentiation of transplantable NPCs from primate ESCs [115]	Zhang SC, *et al.*		Embryoid bodies	bFGF, isolation	NPCs	Rosette formation
US7504257 (**2009**)	ESCs and NPCs derived therefrom [116, 117]	Reubinoff BE, *et al.*	US7011828 (**2006**)	Prolonged culturing at high density on a fibroblast feeder layer	Serum free medium supplemented with EGF and bFGF, isolation	NPCs, or neurons and glial cells	PSNCAM, Nestin, vimentin, Pax-6
US7604992 (**2009**)	Generation of NSCs from undifferentiated hESCs [118]	Reubinoff BE			FGF-1, 2, 6, 8, 9, 17	NPCs, or neural cells	
US7445931 (**2008**)	Compositions and methods for enrichment of NSCs using ceramide analogs [119]	Condie BG, Bieberich E		Embryoid bodies	Ceramide analogs	NSCs	
US7531354 (**2009**)	Neuronal progenitors from feeder-free hESC culture [120]	Stice S, *et al.*		Adherent hESCs in a serum free differentiation medium	bFGF, LIF, sonic hedgehog, retinoic acid, isolation	NPCs, or motor neurons	Nestin (>90%), PSNCAM (-), A2B5 (-)
US7579188 (**2009**)	Oligodendrocytes derived from hESCs for remyelination and treatment of spinal cord injury [121]	Keirstead HS, Nistor GI	US7285415 (**2007**)	Embryoid bodies	bFGF, thyroid hormone T3, retinoic acid	Oligoden-drocytes and their precursors	NG2 proteoglycan (>80%), GalC (>95%), NeuN (-)
US7541186 (**2009**)	Method of generating human retinal progenitors from ESCs [124]	Reh T, Lamba D		Embryoid bodies	IGF-1, Dkk-1, Noggin, isolation	Retinal progenitor cells	
US7795025 (**2010**)	Methods for producing enriched populations of human retinal pigment epithelium cells [125]	Klimans-kaya IV, Lanza R	US7736896 (**2010**), US7794704 (**2010**)	Multilayer adherent cells	Media lacking bFGF, isolation	Retinal pigment epithelium cells	Brown pigment, Pax6 (-), bestrophin (+), CRALBP (+), PEDF (+), RPE65 (+), cobblestone, polygonal, epithelial-like

**Table 4:** Patents Related to Cardiac Differentiation of Human Embryonic Stem Cells.

Publication Number and Date	Title and Ref. No.	Inventors	Division or Continuation of Patent	Initiating Differentiation	Differentiating Agents or Methods	Covered Human Cardiac Cells	Cardiac Markers or Phenotypes
US7611852 (**2009**)	Functional cardiomyocytes from hESCs [128]	Thomson JA, *et al.*		Embryoid bodies	Spontaneous differentiation for 40-95 days, isolation	Atrial-, ventricular-, nodal cardiomyocytes	Electrical activity
US7638328 (**2009**)	Method for efficient transfer of human blastocyst-derived stem cells from a feeder to a feeder-free culture system, and use for myocardial regeneration [129]	Eriksson P, *et al.*		Feeder-free culture, embryoid bodies	Spontaneous differentiation, isolation	Cardiomyocytes	MHC, troponin I, troponin II, GATA4, Nkx2.5, ANF
US7732199 (**2010**)	Process for making transplantable cardiomyocytes from hESCs [130-133]	Xu C, Gold JD	US7425448 (**2008**), US7851167 (**2010**), US7763464 (**2010**)	Embryoid bodies	Activin A, TGFbetaI, IGF II, BMP 4, FGF 4, Insulin, bFGF, PDGF, 5-aza-deoxy cytidine, isolation	Cardiomyocytes and their precursors (>5%)	Cardiac troponin I, cardiac troponin T, atrial natriuretic factor, MHC, GATA-4, HNF3b, spontaneous contractile activity
US7452718 (**2008**)	Direct differentiation method for making cardiomyocytes from hESCs [134]	Gold JD, *et al.*		Adherent solid surface comprising a substrate coated with gelatin or fibronectin	Activin A, BMP-4, isolation	Cardiomyocytes	MHC
US7727762 (**2010**)	Method of inducing the differentiation of stem cells into myocardial cells [135]	Fukuda K, *et al.*		Embryoid bodies	BMP antagonist (Noggin, Chordin, fetuin, follistatin, sclerostin, DAN, Cerberus, gremlin, Dante), isolation	Cardiomyocytes	
US2007-161107 (**2007**)	Differentiation of hESCs to cardiomyocytes [136]	Mummery C, *et al.*		Co-culturing with cells or extracellular media, embryoid bodies	Excreted cardiomyocyte differentiation inducing factor	Cardiomyocytes	
US2008-031857 (**2008**)	Cardiomyocyte Differentiation [137]	Passier R, Mummery CL		Co-culturing (END2 cells), embryoid bodies	Excreted cardiomyocyte differentiation factor, inducing local aggregation	Cardiomyocytes	
US2010-183565 (**2010**)	Induction of hESC derived cardiac pacemaker or chamber-type cardiomyocytes by manipulation of neuregulin signaling [138, 139]	Laflamme MA, Zhu WZ		Conditioned medium-, feeder-, serum-free	Activin A, BMP-4, Isolation	Cardiomyocytes	Nodal/pacemake, atrial/ventricular phenotype

**Table 5:** Patents Related to Pancreatic Differentiation of Human Embryonic Stem Cells.

Publication Number and Date	Title and Ref. No.	Inventors	Division or Continuation of Patent	Initiating Differentiation	Differentiating Agents or Methods	Covered Human Endoderm Cells	Endoderm or Pancreatic Markers or Phenotypes
US7326572 (**2008**)	Endoderm cells from hESCs [144, 145]	Fisk GJ, Inokuma MS	US7033831 (**2006**)	Feeder-free, Embryoid bodies	Activin A, butyrate, retinoic acid, TGF-beta antagonist (Noggin), nicotinamide, EGF, bFGF, betacellulin, isolation	Endoderm cells, insulin secreting cells	
US7704738 (**2010**)	Definitive endoderm [146, 147]	D'Amour KA, *et al.*	US7510876 (**2009**), US7625753 (**2009**)	Manipulating adherent cell density and serum concentration	Nodal, activin A, activin B, Wnt3a, isolation	Definitive endoderm cells	SOX17, HNF3beta, CXCR4
US7541185 (**2009**)	Methods for identifying factors for differentiating definitive endoderm [148]	D'Amour KA, *et al.*			Retinoid, retinoic acid, FGF-10, FGF-2, Wnt3B	Definitive endoderm cells	PDX1, HOXA13, HOXC6, PROX1, HAS, TITF1, CDX2
US7695963 (**2010**)	Methods for increasing definitive endoderm production [149]	Agulnick A, *et al.*			Nodal, activin A, activin B, BMP, E-cadherin antibody, isolation	Definitive endoderm cells	
US7695965 (**2010**)	Methods of producing pancreatic hormones [150]	Martinson L, *et al.*	US7534608 (**2009**)		Activin A, activin B, Wnt3a, SB-431542	Pancreatic endoderm cells, or insulin secreting cells	PDX-1, or response to glucose stimulation
US7772001 (**2010**)	Directed differentiation of ESCs into an endoderm cell [151]	Benvenisty N		Embryoid bodies	NGF, HGF, dissociating to single embryonic cells and culturing as a monolayer	Endoderm cells	
US7585672 (**2009**)	Differentiation of stem cells to endoderm and pancreatic lineage [152]	Odorico J, *et al.*		Embryoid bodies	Selection by magnetic activated cell sorting with cell surface antigens	Endoderm and pancreatic cells	EpCAM
US7763466 (**2010**)	Mesoderm and definitive endoderm cell populations [153]	Keller GM, *et al.*		Embryoid bodies, absence of serum	Activin, isolation	Mesendoderm, mesoderm, endoderm cells	HNF3beta., Mixl-1, Sox17, Hex-1, or pdx-1

## ABBREVIATIONS

bFGF: = Basic fibroblast growth factor
CNS: = Central nervous system
EPO: = The European Patent Office
hESCs or ESC: = Human embryonic stem cells or embryonic stem cell
hNSC or NSC: = Human neural stem cell or neural stem cell
hPSCs: = Human pluripotent stem cells
hSSC: = Human somatic stem cell
ICM: = Inner cell mass
iPSCs: = The induced pluripotent stem cells
IVF: = *In vitro* fertilization
MEF: = Mouse embryonic fibroblasts
NPC: = Neural progenitor/precursor cells
FDA: = US Food and Drug Administration
USPTO: = The U.S. Patent and Trademark Office
WARF: = The Wisconsin Alumni Research Foundation
